# The understanding, application and influence of complexity in national physical activity policy-making

**DOI:** 10.1186/s12961-022-00864-9

**Published:** 2022-05-31

**Authors:** Benjamin P. Rigby, Caroline J. Dodd-Reynolds, Emily J. Oliver

**Affiliations:** 1 MRC/CSO Social and Public Health Sciences Unit, University of Glasgow, Berkeley Square, 99 Berkeley Street, Glasgow, G3 7HR UK; 2 Department of Sociology, Durham University, 32 Old Elvet, Durham, DH1 3HN UK; 3 Department of Sport and Exercise Sciences, Durham University, 42 Old Elvet, Durham, DH1 3HN UK

**Keywords:** Complexity, Systems, Policy, Implementation, Physical activity, Qualitative research

## Abstract

**Background:**

Complexity theory and systems-thinking are increasingly popular in physical activity (PA) research and policy discourse. The impact of this perspective shift, across many sectors, may be underwhelming. We explore why, by focusing on how these concepts are understood and applied by PA policy-makers. This is of particular interest given the challenges of multisectoral interest and poorly defined stakeholder boundaries that are associated with PA promotion. In this study, we critique key elements of complexity theory and consider how it is understood and put into practice in PA policy-making.

**Methods:**

We adopted a complex realist position. Ten semi-structured interviews were conducted with national-level policy-makers from United Kingdom government settings (five civil servants, three politicians, two policy advisors). An inductive thematic analysis was conducted, and managed with NVivo 10 software.

**Results:**

Three overarching themes were constructed to reflect policy-makers’ *uncertainty* about complexity and the application of such perspectives to this policy space, their sense that PA was an *unexceptionable yet unclaimed policy issue*, and their desire for *influence and change*. Participants discussed complexity in contrasting ways. Its meaning was context-dependent and dynamic, which generated uncertainty about applying the concept. Participants also perceived an increasingly diverse but ill-defined PA policy system that spans the domains of expertise and responsibility. Collaborative practices may contribute to a previously unobserved sense of detachment from the systems’ complexity. Nevertheless, participants suggested potentially effective ways to stimulate system change, which require passionate and enterprising leadership, and included varied evidence use, a focus on localised implementation and different ways to connect people.

**Conclusions:**

This research highlighted the importance of extending complexity theory and systems-thinking. While emphasizing the prevalence of these ideas across the PA sector, there is uncertainty as to their meaning and implications. This may prevent their use in ways that enhance PA policies and programmes. Participants conceptualised PA as a tool, which was imposed on the system. While this may support participative decision-making and localised implementation, further research is needed to understand how local systems foster leadership, the practical application of complexity and systems-thinking, and how to support system-wide change in the development and implementation of PA policies.

**Supplementary Information:**

The online version contains supplementary material available at 10.1186/s12961-022-00864-9.

## Background

The benefits of physical activity (PA) are well known, yet globally many people do not undertake recommended levels [1, 2]. In the United Kingdom, 62.8% of adults and 44.9% of children meet aerobic activity guidelines [3, 4]. Recent years have seen increased demand for robust policy action to tackle inactivity [5, 6]. This has been accompanied by growing interest in applying complexity theory in policy at all levels through interdisciplinarity and systems-thinking to respond to the intractability of inactivity [6–10]. This reflects an expansion of complexity-related thinking in public health research more generally [10–12].

However, we argue that despite the proliferation of complexity-based ideas, the impact of this perspective shift has so far been underwhelming and has failed to translate into effective policy action and thus changes in population activity levels. This paper seeks to understand why, by focusing on how complexity is understood in relation to PA by national-level policy-makers in the United Kingdom. We explored how their understanding influences their work and broader efforts to promote PA. In particular, we critique core tenets of complexity theory [13–15] to consider their operationalization “in the field” of PA policy-making.

### What is complexity?

In this paper, complexity is discussed in broad terms and encompasses theories, methods and academic disciplines associated with complexity science and systems-thinking [16, 17]. Complexity science typically focuses on analysing change in dynamic systems over time, while systems-thinking is more concerned with the boundaries drawn around system structures and the interaction of agents (e.g. people and institutions) within the system [18].

The concept of complexity has gained traction in social sciences and policy research over the past quarter-century, with developments in research methods, methodological perspectives (e.g. implications for realism as applied in this paper), and how complexity can inform existing research practices [16]. Recently, there has been a further turn towards critical reflections on how these ideas are used in practice [16, 19–21]. It is generally accepted that complexity focuses on the behaviours and interactions within and between systems, which are largely unpredictable. For example, Barbrook-Johnson et al. [22] summarized key characteristics of complex systems to include:

…their adaptive and dynamic nature, feedback loops, multiple scales, thresholds for change, areas of high and low stability, and open or ill-defined boundaries that can span (sociotechnical) domains or areas of expertise and responsibility. Such features result in systems characterized by tipping points, nonlinearity, emergent properties, and unpredictability. (p. 316)

These features typify United Kingdom policy-making environments [23] and the issues, such as inactivity, for which policy responses are developed [22].

### How policy-makers understand complexity

Complexity in policy-making is poorly defined [14]. Therefore, policy-makers may learn about and discuss it through narratives and metaphors [14, 24, 25]. This results in numerous conceptualisations that may lead to misapplication of terminology and policy tools across different fields when translating conceptual discussions into firm policy action. Furthermore, scepticism about issues of common interest may grow [14, 26, 27].

People typically have an intuitive understanding of complexity in their own field [28]. For example, policy-makers recognise that their work is multicentric or cite the multisectoral nature of PA promotion [23, 29]. However, this understanding is seldom literal (i.e. reflecting principles of complexity science [30]), and usually fails to account for how complexity is perceived elsewhere [31, 32]. Recent research exploring food-energy-water-environment policy evaluation exemplified this [22]. In practice, complexity derived not from scientific theories but from issues of scale, unpredictability and context. The authors concluded that pragmatic framing and communication of complexity were key to support evaluation. However, they elected not “*to ‘judge’ participants’ views against academic definition and debates*” (p. 317). While it is important to understand how complexity is experienced in given settings, the absence of reference to theoretical constructs precludes the advancement of said concepts for broader application in alternative domains.

### How policy-makers navigate complexity

Research has demonstrated complexity’s influence on policy-makers’ actions and their ability to reflect on these [14, 24, 27]. People often behave so as to survive amid perceived exhaustion, diminished control or inability to address issues single-handedly [32–34]. However, three strategies may help policy-makers navigate complexity more effectively: (i) harbour realistic expectations about policy aims and impact, not least due to bounded rationality (i.e. policy-makers’ cognitive and information-gathering abilities are limited) [14, 24]; (ii) adopt longer-term perspectives that embrace experimentation and innovation [14, 35, 36]; and (iii) act in the system’s interests (i.e. publicly test ideas, and avoid self-referential behaviour or programmes that prioritise the provision of goods and services [32–34]). However, numerous factors may impede these strategies and render policy action difficult.

### The present research: responding to issues in United Kingdom PA policy

Efforts in the United Kingdom to address complexity in PA promotion may have been hindered by an overemphasis on natural science-orientated evidence to inform policies, which are often ambiguous or packaged in marketised terms [37–39]. This reflects a tendency among policy-makers to prioritise linear evidence models or act cautiously amid complexity, where policy change may be politically and financially costly [33, 40]. Even when change occurs, learning is naturally slowed by complexity [41]. Self-organization and negative feedback mean systems may revert to their original states [42, 43].

Despite this nascent understanding of how complexity may influence policy-makers’ actions, existing research in this area has been largely conceptual or anecdotal. There lacks critical reflection on how complexity theory’s principles that underpin the growing movement towards systems-thinking in health promotion are understood and navigated in policy-making. This precludes theories about how understanding influences cross-government approaches to public health.

Furthermore, the lack of domain-specific knowledge relating to PA warrants examination. PA is a particularly interesting context for operationalizing complexity principles, for several reasons. First, the issue and its influencing system are cross-sectoral with poorly defined boundaries. Despite insufficient government strategies that reach beyond health sectors [5], the broader interdependence emanating from formal and informal partnerships required to tackle inactivity is creating “accidental” PA policy-makers [44, 45]. Second, inactivity is a long-standing issue that has proved challenging to impact. Third, the SARS-CoV-2 pandemic has had a significantly negative impact on PA among the United Kingdom population, with an estimated 3 million fewer adults classified as active in November 2020 compared to 12 months earlier [46, 47]. However, amid a renewed policy emphasis and heightened awareness of PA’s benefits in the United Kingdom, a critical window for change may be opened [48–50]. Consequently, and building on emerging works, our research explored the processes, values and experiences of PA policy-makers, and how they collaborated and with whom to foster positive system change.

## Methods

This research was designed and reported according to the consolidated criteria for reporting qualitative research (COREQ) [51] (see Additional file 1). It was underpinned by complex realist ontology (i.e. a framework that helps one understand how society comprises interacting systems with objective properties and causal mechanisms that generate specific events and experiences [13, 52]). These events and experiences were understood through two lenses: (i) participants’ empirical observations, and (ii) the analysis. This analysis was informed by the complex realist perspective, in particular its post-disciplinary characteristics that enabled the application of numerous theories to make sense of the data [13]. Furthermore, it orientated our examination of the data to construct a truthful narrative from among varied cases, through which mechanisms were inferred and observations were explained with reference to key features of complex systems.

### Study design

Semi-structured interviews were conducted face to face (*n* = 1) or by telephone (*n* = 9). Telephone interviews helped overcome participants’ scheduling constraints. They produce data comparable to face-to-face methods and facilitate participation among élites who may otherwise be difficult to engage [53].

### Participants

Initially, policy documents and websites were used to identify policy-makers whose remit included PA. Participants were selected purposively and invited to participate by email, letter or social media. To maximise recruitment, follow-up correspondence was sent at 2 and 5 weeks after the initial invitation.

Thereafter, snowball sampling [54] was adopted and potential participants were approached as above. Data saturation was considered immaterial for conducting a thematic analysis on data from a niche targeted group [55, 56]. Recruitment ceased when attempts to engage identified individuals were exhausted.

Thirty-eight individuals were invited to participate (10 offered no response, 16 declined, two failed to return consent forms). Where reasons for non-participation were offered, one individual had changed role, one’s remit contained little PA, and others cited time constraints. Participants were drawn from across government and associated organisations, as well as one university. See Additional file 2 for further information.

### Procedure

The research team developed the interview guide to facilitate discussion, with reference to literature on complexity, policy-making and PA promotion (see Additional file 3). The guide was reviewed by a non-participating civil servant, and then piloted with three individuals with previous policy-making experience, who participated no further. As a result of the pilot process, the guide’s structure and language was adjusted to be more accessible to policy-makers working in this domain. Further iterative changes were made during the project to reflect how policy-makers discussed concepts and how particular information was best accessed. By default, participants did not receive the guide before interviews. However, three requested it. One stated that they had prepared responses with colleagues.

All participants provided written informed consent. Data were collected between July and October 2018. All interviews were conducted by BR. Prior to commencing, participants completed a short demographic questionnaire and BR briefed them about the project and the researchers’ interests and assumptions, invited questions and obtained final verbal consent. Interviews were audio-recorded and began with general conversation to build rapport. Notes were taken throughout. During telephone interviews, BR remained attentive to intonation to recognize cues more easily seen in face-to-face settings. Towards the end, participants were invited to ask questions. This sometimes prompted further data generation. Thereafter, participants were debriefed about data use and their continued involvement. Interviews lasted between 17 and 69 minutes (average 35), which was a function of the difficulties in accessing policy élites for interviews [53], but is nonetheless indicative of their engagement with the research environment. In this context, engagement was deemed meaningful and offered sufficient data to examine the salient patterns across a collated data corpus.

Audio recordings were transcribed verbatim. Identifying information for individuals and some organisations was removed. Participants received a copy of their transcript and were invited to comment (two did so), and received monthly update emails throughout data collection and analysis.

### Analysis

Data were analysed using an inductive thematic approach [57] to identify salient patterns across the dataset without a predetermined coding framework. QSR NVivo 10 software assisted data management and analysis. BR and EO immersed themselves in the transcripts and made initial notes. Line-by-line semantic and latent coding was conducted by BR. Codes were iteratively consolidated into a final coding framework containing 26 free codes. The dataset was recoded. These codes were then organised into candidate themes, which were reviewed against the coded data extracts (BR) and then the entire dataset (BR, EO, CDR). Through discussion, the research team refined and combined these into three overarching themes and seven lower-order themes. Diverse cases were also included to reflect the range of views raised. Participants received a findings report, to which five offered comments. The research team considered and incorporated these as appropriate.

## Results

### Participant characteristics

Ten policy-makers (eight men, two women; aged 34–69 years) participated in interviews. Participants each chose a descriptor for use below. These were “civil servant” (CS), “policy advisor (PA)” or “politician (P)”. Additional characteristics have been withheld to maintain anonymity of this niche group.

### General themes

**Fig. 1:**
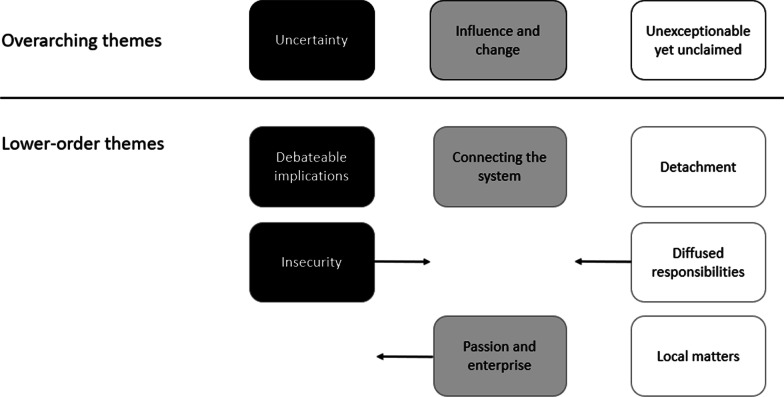
Thematic hierarchy showing overarching themes and lower-order themes (arrows denote potential relationships between themes)

Three overarching themes concerned how participants understood complexity. How they understood the PA system, and the resultant impacts on their practice were constructed from the data: *uncertainty*, *an unexceptionable yet unclaimed policy issue*, and *influence and change*. Figure 1 provides an overview of these and associated sub-themes.

#### Theme 1: Uncertainty

Among participants there was collective “uncertainty” about the concept of complexity, manifesting in two ways. First, participants’ varied and inconsistent attempts to define complexity reflected their understanding. Second, participants were divided as to how they perceived that complexity influences their efforts to address physical inactivity and public health issues more widely. This “uncertainty” was perceived to relate to how the concept is learned about and subsequently transmitted (or not) across the sectors of government and allied agencies whose remits include the development and implementation of PA policy.

Not without exception, participants expressed some awareness and “*crude understanding*” (CS2) of complexity and systems. However, this understanding was diverse and expressed in 12 different ways across the dataset. Complexity was conceptualized as follows: the absence of simple solutions; different contexts; holism; logic models; long-term processes; moving at scale; multifaceted; multilevel; thinking in the round; vested interests; a web of integration; and whole systems. Sometimes, these reflected scientific principles of complexity, such as those outlined by Rutter et al. [56] (e.g. multiple interconnected elements that influence PA and the need for iterative programme testing and adaptation). Nevertheless, as previous research has suggested [14], participants tended to convey their understanding through metaphors given the absence of a common language. For example, Civil Servant 5 commented, “*You mentioned Foresight, and you’ve seen that map, it’s like spaghetti junction*.” Regarding policy, others acknowledged that “*there’s no silver bullet*” (PA1) and “*you need what some describe as a smörgåsbord of options*” (PA2). Some participants described the cross-sector interactions that may ultimately facilitate the development of a common language:

*Whole-systems approach is certainly the language of colleagues in DHSC [Department of Health and Social Care] and PHE [Public Health England]. They certainly talk about and think about it now. We had a recent session with Sport England. They were talking in that language as well […] through conversations with DHSC has helped me understand it as well.* (CS4)

Contrastingly, others distanced themselves from these concepts, demonstrating some resistance to their increased prevalence in policy-making. Complexity was considered “*scientific […] a more mathematical word*” (CS3), its use exemplifying “*a tendency to over-apply academic practices*” (P1) to the real world of PA practice.

Complexity was often perceived to be someone else’s concept. The way in which participants discussed how complexity was learned about through sharing experiences and practices, yet nobody claiming ownership over the complexity, reflects how complexity’s meaning continually evolves from scientific, political and practice-orientated perspectives [24, 59]. However, this fluidity may lead to misapplication, scepticism and uncertainty [14, 27], which were evident in our study.

#### Sub-theme 1.1: Insecurity

Participants seemed to lack confidence and express uncertainty in their understanding of complexity. Furthermore, our findings suggest that while policy-makers engage with ideas, such as complexity, that may be unfamiliar to them [60], their doubts indicated a potential difficulty in identifying how complexity works for them. This may inhibit stakeholders’ attempts to create effective systems-based solutions for PA.

For example, reflecting on their understanding of complexity, civil servant 3 commented, “*it doesn’t mean anything to me, I’ve been thinking about this*.” Participants’ apparent willingness to learn, however, was related to how they appraised their knowledge and skills amid predominant discourses:

*They were talking in that [complexity] language […] and you might not entirely know what someone’s talking about but you ask the question, “I’m sorry, what did you mean?”* (CS4)

This extract further emphasizes how interactions influence policy-makers’ conceptual considerations. Adopting “*evidence, theories and ideas, testing and refining those in different kinds of environments*” (CS1) was considered pivotal for advancing PA promotion.

Several participants were uneasy discussing complexity, speaking in hesitant or speculative terms:

*Oh blimey, now you’re testing me, erm, I think a whole-system approach, it would be a way of looking at things in a holistic way, erm [emphasis added].* (CS2)

Despite their insecurities, participants’ understanding was at least partially aligned with how complex policy problems are discussed in academic literature, for example the importance of “*recognizing these wicked issues*” (PA2) [43, 61]. Nevertheless, other interpretations of complexity principles reflect the potential pitfalls that may arise if policy-makers lack the confidence to apply theories as intended. For example, while complexity necessitates an element of holistic thinking, it is not in itself synonymous with holism [13].

While it has been suggested that “insecurity” may reflect policy-makers’ discomfort at new working practices [62], our data suggest an environment open to change. Rather, the discomfort arose from the participants’ sense of the *debatable implications* of complexity in policy-making.

#### Sub-theme 1.2: Debatable implications

Participants were divided in their beliefs about how useful complexity theory is to their understanding of public health issues and the way that they conduct their work. Sceptics cited how the long-term approaches that complexity theory advocates are often incongruous with the short-termism of politics, public policy and funding opportunities. Nevertheless, others felt that complexity offers a way to consider all of the potential influences of population PA. Some participants believed it was useful, as “*trying to achieve something substantive, you do have to look at how the thing works as a whole*” (CS1). Complexity supported discussion around systems-thinking. Despite one participant stating confidently, “*I think there is a shared view that we have to use a whole-system approach*” (PA1), others recognized its limitations in current circumstances:

*I’m clear with the aspiration, I’m not sure how well it works […] whether it’s a good thing to do is not really controversial, of course it is […] the question is, how realistically do you get our departmental system to think in a whole-system way? […] particularly in a political system that is very short-termist.* (P3)

Such comments suggest that rather than questioning themselves, policy-makers expressed insecurity towards government operations, such as short-term policy goals, that commonly inhibit change [62]. For example, as expressed by most participants, “*one of the challenges is about people having the confidence and willingness to sign up for the long term*” (PA2). This suggests scepticism about whether complex approaches are suitable given the short-term dynamism of government and the unlikelihood of immediate reward for policy-makers advocating substantive change.

Contrary to the positive and ambivalent views raised, others constructed complexity to be a negative concept with little utility:

*It feels like a cop-out […] the whole system has to matter, but what does that add that any other analysis doesn’t?* (P1)

*I think complexity doesn’t really mean much. I would much rather we used the word “complicated”.* (CS3)

Such beliefs may highlight the aforementioned difficulties in applying complexity given its disparate meanings [14]. Furthermore, framing issues as complicated may prevent recognition of systems’ emergent and interactive properties. This has important ramifications for how policy-makers consider their place in relation to issues like physical inactivity.

In sum, although policy-makers understood various features of complexity, understanding was seldom literal [30]. Complexity remained contested in both meaning and utility. This has particular implications for PA promotion where increasingly policy-makers are from varied, nonspecialist backgrounds [44].

#### Theme 2: PA is an unexceptionable yet unclaimed policy issue

This second overarching theme captures the way that participants understood complexity in relation to PA specifically. Participants drew comparisons between inactivity and other public health problems that they perceived to be complex, to articulate the need for particular working practices (e.g. multilevel collaboration and cross-sectoral policy alignment). However, these comparisons and practices also highlighted potentially problematic approaches to addressing the complexities of PA, leaving a sense of unclear ownership in this policy area.

Participants believed that physical inactivity “*is a complex problem, [but] not uniquely complex*” (CS1). It was deemed similar to issues like obesity and type II diabetes due to having multilayered determinants [45, 63]:

*You very quickly realise that everyday decisions are influenced consciously and unconsciously by family, community, society, government and, in some senses, international context.* (PA1)

However, perceived similarities gave rise to an apparent sense of detachment from the complexity of both inactivity and the policy-making environment. Policy-makers intermittently considered inactivity, more as outsiders seeking to change the system, but seldom took ownership of the issue, as their responsibilities and roles were obfuscated by the complexity of the policy environment.

Perceiving inactivity as similar to other public health issues may be useful, if this facilitates the transfer of knowledge and programmes to address it. However, politicians questioned whether, unlike other issues, PA promotion generated political capital:

*It’s about doing a lot of the small things, and that makes it sort of less sexy […] having a policy proposal that says everybody should do a bit more isn’t very exciting.* (P1)

Inactivity was seen as uncontentious, and policy-makers looked towards more contestable issues through which inactivity may be considered (e.g. “*obesity is the best bet*” [P2]). While aligning PA policy with other objectives recognises the benefit of cross-sectoral approaches to public health [64], and participants seemed to realise their interdependent endeavours, it is unclear to what extent they understood their interactions with wider elements in the system, in a manner consistent with the specific implications of interaction that complexity theory proffers.

#### Sub-theme 2.1: Diffused responsibilities

Interdependence and the consequent systems-thinking was a major perceived similarity between inactivity and other issues. This resulted in a perception that responsibility for PA policy is increasingly diffuse. Supporting this argument, participants cited experiences of multicentric policy-making and governance, cross-sector working, and reconciling disparate stakeholder views. However, these may be challenging:

*It’s about working with a range of partners and perhaps trying to pool budgets and resources […] and it is wider than just departments because it’s the community and voluntary sectors, the non-statutory bodies and so on, that are perhaps closer, more on the ground.* (CS5)

*It’s just my experience, getting departments to work together is easy in principle, it’s the practice of how do you get them to sign up to something that is cohesive, that commits themselves and the people they are responsible for down locally.* (P2)

Nevertheless, cross-sector interdependence was considered to be positive. Policy advisor 1 acknowledged, “*It does well for us to be able to cross-pollinate between different modalities and different bits of the sector*”. In particular, participants “*recognised that decisions on these things are devolved down*” (CS1), and advocated for the importance of effective local systems. This reflected an increased emphasis on devolution and democratic renewal [65], as well as responsibility for the enactment and implementation of PA policy.

#### Sub-theme 2.2: Local matters

Participants deemed it necessary to “*create enthusiasm for*” (P2) localised policy responses, which are important amid complexity [14]:

*A key playing area about how effective policy is, you can set a national strategy, but unless you’ve got local bodies […] it’s kind of how they apply that locally.* (CS4)

Local governance was believed to provide flexibility. Being “*light-years away from that [top-down] approach*”, it enabled “*a lot of experimental stuff*” (CS4), such as new contextualised programmes and decision-making structures for PA promotion. This approach may explain why, contrary to previous research [40], participants did not feel “*a sense of frustration or anger when local authorities aren’t promoting [PA] as much as the government would like*” (CS2). Indeed, there was recognition that “*sometimes it’s quite a lonely place*” (CS1) promoting PA with minimal local resources.

However, national-level policy-makers’ capacity to respond to local complexities seemed limited. Although “*national programmes are tested at one or a couple of local levels*” (CS1), a more concerted effort to support necessarily localised implementation may be required [66]. For example, the 2011 United Kingdom PA guidelines [67] “*did not have an implementation plan*” (CS3). “*While not unique to physical activity, the challenge is moving beyond just writing [policy] to then implementing*” (PA1). One way in which policy-makers may attempt to bridge the national–local implementation gap is by drawing on a variety of evidence, through political-, scientific- and practical implementation-focused lenses [59]. This means that to make sense of complexity, science from across the hierarchy of evidence is necessary but not sufficient. It is important to consider other types of evidence such as practical know-how about influencing policy and politics, and delivering and evaluating programmes.

#### Sub-theme 2.3: Evidence and implementation

Participants expressed the view that the complexity of PA means that they needed to seek different forms of evidence to help bridge the gap between policy-making and the local systems in which national policies are implemented. Nevertheless, it is important to remember that policy-makers receive copious amounts of information and have bounded rationality [68]. Consequently, they perhaps “*don’t have the time to delve into the complexity*” (CS4), as researchers were expected to do. While most policy-makers seemed “*committed to the evidence-based policy-making*” (P1), there were challenges. Sometimes they had to “*take a hunch*” (CS3), “*make the policy then go and find the evidence*” (P2) and tackle normative pressures, for example, “*[civil servants’] natural, understandable prejudices against the idea made the whole system very resistant*” (P3). In this context, the participant discussed policy-makers’ stereotypical views of certain types of PA and its participants.

Furthermore, participants did not always seem clear in their understanding of how different forms of evidence intersect:

*We have to look at whether or not the pragmatic delivery of physical activity informs academic research, or does academic research then inform what we should be doing.* (CS1)

*Sometimes the knowledge translation from academic to practical implementation can be difficult.* (CS5)

Increasing localised evidence production among ever-diversifying stakeholders, coupled with a “*move towards place-based thinking*” (PA2), may render national policy-makers’ position in the system somewhat unclear and detached.

#### Sub-theme 2.4: Detachment

Our data suggest that participants disassociated themselves from complexity. That is, they did not necessarily see themselves to be constituent elements of the system. The system was intervened with and then withdrawn from, a sense that there would be a “tipping point” where PA no longer needed deliberate promotion efforts and “*we can walk away, job done*” (PA1). This is important because detachment from the issue and its complexity, wittingly or otherwise, imposes barriers to achieving policy goals, as one cannot stand outwith a system they seek to change [15].

Physical inactivity was “*frankly marginalised*” (P1) and was “*everyone’s business but nobody’s responsibility*” (CS5). For example, the issue was seemingly passed between different system agents:

*If we regard this as a public health activity, the responsibility has been transferred to local government, so I would push it back to the director of public health and well-being boards.* (P2)

That the issue was passed between agents and institutions may be indicative of policy-makers simplifying their environment and absolving themselves of responsibility, or a variation on the tragedy of the commons [69–71]. However, it appears as likely that participants struggled to understand how systems interact, both internally and with other systems. To overcome a resultant “*perceived lack of coordination*” (CS5), stakeholder-led strategies may help agents “*recognize their roles in creating a more active population*” (PA2). But one participant cautioned:

*One of the challenges of a whole-system approach is it requires you to be able to step back and go “actually, the best person to do this sits over there in a different sector and is not me.* (PA1)

It is necessary to consider that the broader context (e.g. competing priorities, economic change, political processes) and organizational complexities, emanating from “*policies that fit between numerous departments*” (CS2) and nongovernmental sectors, may partially explain why PA is not always a policy priority [5]. However, the evidence presented here suggests something further, and has important implications.

It is known that system-wide identity (i.e. both the individual agents’ sense of role and place within the system, and how these individuals collectively understand their position), which is perhaps unclear in the PA domain, motivates agents towards goals [72]. Furthermore, by characterising physical inactivity as a discrete entity separate from the structural properties and causal mechanisms of the system by which the problem is generated, this reifies and legitimises the conceptualization of PA as a technology in the sociological sense [73], and therefore its subsequent use.

#### Theme 3: Influence and change

This theme characterises how policy-makers’ perceptions of complexity and inactivity affected their work in the face of enduring systemic barriers. Some policy-makers expressed feelings of control and empowerment that may have arisen from the autonomy of non-departmental organisations [74], statutory instruments or agents’ behaviours:

*It has a lot to do with personality. What you had was [a] senior clinician who wasn’t a single top person and therefore could jump across and use the network to find synergies and opportunities.* (PA1)

However, several policy-makers questioned their influence, noting that “*not all policy levers are held by government*” (CS2) and that PA “*prevalence has remained stubbornly the same*” (CS3). They were left looking to others, querying “*where’s the leadership?*” (P2). This perceived lack of influence may partially explain existing and potentially detrimental reactions to complexity among PA promoters, which are discussed in turn.

Three possible issues were present in the data: (i) most participants cited short-termism [36]; (ii) two participants emphasized PA in marketized terms [33]; and (iii) some displayed self-referential behaviour and apportioned blame to other elements of the system [32]:

(i) *Governments come and go* (CS2). *If you’re looking at a whole-system approach, that unit of time is nothing* (P3).
(ii) *I think it’s selling a product to people who do not want it* (CS3).
(iii) *I’m not sure public health like sports, they’re very blinkered* (P2).

This perceived lack of influence, short-termism and the prioritisation of departmental and ministerial priorities perhaps explains why “*this silo thing is quite normal*”. However, policy-makers recognised that these behaviours are not necessarily beneficial, and articulated a solution-focused mindset towards connecting the system.

#### Sub-theme 3.1 Connecting the system

Connecting the system to address inactivity was important to the participants. While some thought “*there’s no effective lobby […] and so you see a very dissipated effort*” (P2), others discussed examples of effective advocacy coalitions and political entrepreneurship:

*A lot of lobbying by organisations and other cycling groups that led to the infrastructure bill being amended […] it’s a credit to them that they managed to influence legislation in that way.* (CS4)

However, without consistent alliances or specific issues to lobby against, participants sought other ways to bring people together.

Because PA, through processes of detachment, had come to be perceived by participants as a technology (i.e. techniques, processes and objects used to provide services and connect people), policy-makers “*tried to weave physical activity into every bit of policy*” (PA1) to overcome silo working. In this way, PA was, in complexity terms, deemed to be the primary boundary-spanner, rather than any particular people or organisations:

*Inputting physical activity into commissioning contracts with other government departments and that kind of stuff. So where would you look at really embedding physical activity into key systems.* (CS1)

Using PA as a tool is inherently political, but commonplace in policy-making and practice [75]. However, this strategy did not appear to galvanise systems, as interdependence between parts of the policy system remained challenging. Participants alluded to three potentially more effective mechanisms: “*good working relationships*” (CS3) between different parts of the system (e.g. regular and mutually beneficial dialogue), “*more investment*” (CS1) in PA policy programmes, and political leverage [e.g. “*we got agreement from the speaker’s office*” (PA1)]. Furthermore, there was one additional feature that participants felt strongly about: the need for leadership to cut across complexity and the intricacies of PA promotion.

#### Sub-theme 3.2: Leadership

Two forms of leadership were evident in our data. First, policy as leadership. Broadly, policy is the decisions or actions adopted by agents to accomplish goals [76]. Some policy-makers felt they could “*pull people together*”, although it may be necessary to “*force them to*” with cross-departmental agreements (P2). For example:

*What we’ve got is the ability to convene and be technical experts and advisors. It’s pretty unique actually, and it has created that glue at a national level, which has supported and enabled the glue at a local level […] we can play that brokerage across the system […] bringing people together in neutral territory.* (PA1)

Second, participants stressed the importance of individual leadership:

*Strengthening the reliance on individuals in key positions to move the agenda forward, it is one of the most fundamental steps when addressing complexity. Having a couple of people in aligned agencies can significantly move forward the agenda.* (PA1)

Some felt that “*it was very minister-dependent*” (P3) or at least required leadership “*at a very senior level that will filter down*” (CS1), for example leaders in the United Kingdom’s National Health Service and DHSC. In addition, there was recognition that local leaders are also necessary:

*We have a number of new mayors […] who are responsible for lot of key transport decisions in their areas. Andy Burnham in Manchester, for example, particularly helped by his active travel commissioner, has really been prioritizing investment in active travel.* (CS2)

Further examination of the data highlighted two key features of PA leaders that characterised this study’s participants.

#### Sub-theme 3.3: Passion and enterprise

Diminished perceptions of control amid complexity may prevent policy-makers from publicly testing new ideas, as they save face or assert their dominance [34, 77]. However, our study portrayed participants as open to new ideas and experimentation, as the following example demonstrates:

*And of course the policy solutions to that are so different to those that have been historic, which have been, “Well let’s just improve the roads and they’ll be nicer to cycle on.” Well actually, what happens if you don’t want to cycle on the road […] what’s the role of e-bikes and stuff like this?* (CS4)

This willingness to adapt is important for navigating complexity in policy-making [35]. Further, throughout discussions, participants demonstrated a desire and “*their hope we can*” (CS3) progress PA promotion. Given that most participants were somewhat active themselves, this passion may emanate from personal experience and being “*people who get it*” (P3). Nevertheless, policy-makers’ ambition to reach out to local bodies and embed effective PA policy programmes nationwide, “*by sharing best practice and schemes that have been successful*” (CS2), was evident.

## Discussion

To our knowledge, this study was the first to explore how national-level PA policy-makers from across government in the United Kingdom make sense of and navigate complexity. The findings highlighted the contested meaning and implications of complexity, raising questions about its practical utility in PA, policy-making and elsewhere [75]. Effective application of complex-based understanding was hindered by detachment (i.e. understanding an issue as complex but not one’s part in the complex system), the increasing complexity of the policy-making environment itself and the failures of subsequent diffused leadership in particular. Despite this, we identified causes for optimism including an openness to pragmatic and experimental approaches, emergent opportunities to foster change, and recommendations that exceed previous framing-based arguments (i.e. different ways of categorizing conceptualizations of complexity) [22], which may support a better balance between complexity theory and its practical application to PA policy.

### Key findings and implications for complexity theory

Our findings support previous research in several ways: (i) complexity is seldom understood or used literally [22]; (ii) policy-makers reflexively engage with evidence [59]; (iii) metaphors influence complexity’s dynamic meaning [14, 24]; (iv) anticipated reactions to complexity are evidenced by the participants’ practices [33, 69, 71]; and (v) increased local co-evolution and adaptation of policies is occurring and desired [33, 78].

These findings reinforce observations that policy-makers have no shared language of complexity and often use metaphors [14]. As a mechanism [79], language creates an understanding of complexity and enables this to transcend common discussions [80]. Furthermore, the current results suggest that a shared understanding cannot be engineered; rather, it may develop, through dynamic social interactions and communities of practice, which may also help identify policy-makers’ roles and address issues of ownership. To some extent, understanding may be emergent [81]. The dynamic, contextually determined meanings of complexity discredit previous notions of conceptual purity [82–84].

Echoing recent concerns [12], our findings also uncover the increasing, if inconsistent, way in which complexity science principles are interpreted in relation to PA policy-making. Complexity theory alone is unlikely to sufficiently address the identified knowledge–implementation gap and better inform public health decision-making. The limitations of complexity and systems-thinking require examination [75].

### Key findings and implications for understanding policy-makers’ decision-making

Decision-making involves both rational or irrational skills and aptitudes [85]. Amid complexity, policy-makers may tend towards irrationality (e.g. emotions, beliefs, familiarity) for prompt decisions [68, 86]. Contrastingly, our findings showed that, despite complexity, PA policy-makers considered and prioritised rational decision-making processes wherever possible (e.g. evidence-based, goal-orientated responses).

Our results show how responsibility shared across the PA system encourages collective and interdependent decision-making. As participants accumulated information, solving the problems facing them from various sources relates to the idea of participative decision-making [87]. By being open to new ideas and agents, and carefully balancing the inherently different interests this creates, this helps policy-makers navigate complexity [88]. However, the “detachment” also associated with diffused responsibilities raises important considerations. It is unclear to what extent a sense of belonging is a necessary component of successful participative decision-making, as motivational need theories may suggest (e.g. [89, 90]). Further study on collective identity is warranted.

Numerous barriers to evidence-based decision-making persist, for example fiscal constraints, short-termism, competing pressures and insufficient leadership [91, 92]. Despite these, participants exhibited behaviours suitable for decision-making where, as in PA policy, failure is common [93]. Policy-makers were willing to be pragmatic, experimental and pluralistic.

Participants acknowledged that more can be done to address gaps between researchers and decision-makers. Our findings suggest that the development of efficient, complexity-sensitive evidence, tools and processes for policy-makers to employ may support the identification of who should respond to evidence, as well as negating factors associated with irrationality, such as time pressures and increased ambiguity [94].

Lastly, previous research on the influence of key individuals in public health decision-making has focused on their common-sense or expert judgements, or how they filter evidence [92]. We extend this knowledge base by highlighting the roles individuals in key positions play in shifting or advocating for particular PA policy agenda, often in cross-sector alliances in order to address complexity.

Furthermore, theoretical advances in complexity propose more diffused models of leadership [95]. In some cases, power and leadership may become so diffuse that it is difficult for policy-makers to recognize who or what constitutes the necessary components of policy change. This may have contributed to our participants’ sense of absent leadership. While they recognized the importance of system-wide leadership, the findings suggest a desire among policy-makers for visible leaders within a top-down framework, emphasizing a role for government and other key organizations in PA policy-making.

### Key findings and implications for understanding the policy-making process

Policy-making analyses encompass policies’ context-specific meanings, as well as decisions and actions adopted to achieve particular outcomes [76, 96]. Our findings demonstrate how, to foster a joined-up response between small departmental teams and increasing numbers of agents beyond government, the inactivity issue is woven into different policies. Through this wilful act of policy-makers to encourage others to recognize the interconnected nature of inactivity and encourage collaboration, policy acquires a semblance of the system-connecting leadership which participants perceived to be elusive (i.e. policy creates bridges between different parts of the system).

Previous research has typically not considered leadership this way, and rather has typically focused on the behaviours and competencies of boundary-spanning individuals [97, 98], which were reinforced in our findings. It is unclear how additional mechanisms for connecting systems (e.g. implementation frameworks or legislated targets to generate buy-in) may support systemic changes, given the short-termism of policy and politics our participants acknowledged.

Furthermore, our findings reflect various elements of policy process theories that identify the importance of coordinated advocacy in punctuating periods of stability with policy change [99–101]. However, they also add specificity that may support PA advocates to engage in policy-making. Participants acknowledged that increasing PA through policy will take time. Consultation, lobbying through networks, and fostering strong relationships with regular interactions are plausible ways to effect quicker policy change, and prepare PA policy responses for opportune deployment.

### Study limitations

Despite extensive recruitment efforts, participants did not engage from all government departments with a PA-related remit (e.g. sport, education or environment). Policy-makers’ views may differ in these fields, and warrant future examination due to their strong policy influence in this domain. Furthermore, we could not consider how experiences differed between organizations and job roles, due to a lack of variation. Future comparative analyses may expose notable political-, ideological- or power-related dimensions related to the development and implementation of policy. Finally, as complex systems evolve, it will be important to continue exploring PA policy systems and the actions of policy-makers over time.

## Conclusions

While complexity science concepts permeate the PA and public health sectors, their meaning and implications continue to be contested, undermining their practical use. In addition, policy-makers’ detachment from the complex system within which they are operating raises practical and ethical questions about ownership and accountability, as well their capacity to effect system change. Against this backdrop, the importance, and absence, of the types of leadership and other mechanisms that may support systems represents a real opportunity. If we can understand how to enable, rather than merely engage, stakeholders from across the system in response to the dynamic opportunities for change that a complex system provides, we can enhance the prospect of effective PA promotion.

## Supplementary Information

**Additional file 1:** COREQ checklist.

**Additional file 2:** Statement of sampling and recruitment.

**Additional file 3:** Interview guide.

## Data Availability

The datasets used and analysed during the current study are available from the corresponding author on reasonable request.

## Abbreviations

PA: Physical activity
DHSC: Department of Health and Social Care
PHE: Public Health England
