# Supplementary material for: The understanding, application and influence of complexity in national physical activity policy-making
Source: Health Res Policy Syst. 2022 May 31;20:59. doi: 10.1186/s12961-022-00864-9 (PMC9153223; doi:10.1186/s12961-022-00864-9)
Supplement: Supplementary file 1 — Additional file 1: COREQ checklist. [file 12961_2022_864_MOESM1_ESM.docx]

# Additional file 1 – COREQ checklist

| **Section/topic** | **#** | **Checklist item and response** |
| --- | --- | --- |
| **Domain 1: Research team and reflexivity** | | |
| Personal characteristics | 1 | ***Interviewer/facilitator – which author/s conducted the interview or focus group?***  See methods section (procedure). |
|  | 2 | ***Credentials – what were the researcher’s credentials?***  See responses to item numbers 3-5. |
|  | 3 | ***Occupation – what was their occupation at the time of the study?***  At the time of writing BR was a full-time funded PhD candidate in the Department of Sociology at the University of Durham, UK. |
|  | 4 | ***Gender – was the researcher male or female?***  BR is a male researcher. |
|  | 5 | ***Experience and training – what experience or training did the researcher have?***  BR had considerable qualitative research experience and postgraduate-level training prior to conducting the interviews, including a Masters degree in *Social Research Methods (Social Policy)*, a UKRI approved core doctoral training module in *Qualitative Methods in Social Science*, and an *Introduction to Qualitative Interviewing* course from the UK Social Research Association. BR had previously published peer reviewed qualitative research articles on physical activity policy. |
| Relationships with participants | 6 | ***Relationship established – was a relationship established prior to study commencement?***  It was infeasible to establish a close relationship with participants prior to interviews given their ongoing commitments. |
|  | 7 | ***Participant knowledge of the interviewer – what did the participants know about the researcher (e.g. personal goals, reasons for doing the research)?***  Participants were briefed from the point of recruitment that BR was to be conducting the interviews as part of his PhD studies. Invitations to participate contained a detailed participant information sheet that introduced the researchers, the project’s importance and what participation entailed. |
|  | 8 | ***Interviewer characteristics – what characteristics were reported about the interviewer/facilitator (e.g. bias, assumptions, reasons and interests in the research topic)?***  In addition to the information outlined against item 7 above, see methods section (procedure). |
| **Domain 2: Study design** | | |
| Theoretical framework | 9 | ***Methodological orientation and theory – what methodological orientation was stated to underpin the study (e.g. grounded theory, discourse analysis, ethnography, phenomenology, content analysis)?***  See methods section (including analysis). |
| Participant selection | 10 | ***Sampling – how were participants selected (e.g. purposive, convenience, consecutive, snowball)?***  See methods section (participants). |
|  | 11 | ***Method of approach – how were the participants approached (e.g. face-to-face, telephone, mail, email)?***  Participants were approached by email, letter or social media. |
|  | 12 | ***Sample size – how many participants were in the study?***  See results section (participant characteristics). |
|  | 13 | ***Non-participation – how many people refused to participate or dropped out? Reasons?***  See methods sections (participants). |
| Setting | 14 | ***Setting of data collection – where was the data collected (e.g. home, clinic, workplace)?***  Data were collected either by telephone (whereby the participant was at home or in an office, and the researcher was at home) or face-to-face in a private office. |
|  | 15 | ***Presence of non-participants – was anyone else present besides the participants and researchers?***  No third parties were present in private offices, although background noise during one telephone call suggested other individuals were present in the workplace. |
|  | 16 | ***Description of the sample – what the important characteristics of the sample (e.g. demographic data, date)?***  See results section (participant characteristics). Considerable information was withheld to protect anonymity of the sample. |
| Data collection | 17 | ***Interview guide – were questions, prompts, guides provided by the authors? Was it pilot tested?***  See methods section (procedure). Topics included: challenges of PA promotion and cross-sector collaboration, complexity and whole systems, and evidence-informed policy. |
|  | 18 | ***Repeat interviews – were repeat interviews carried out? If yes, how many?***  These were one-off research interviews. No repeat interviews were conducted. |
|  | 19 | ***Audio/visual recording – did the research use audio or visual recording to collect the data?***  See methods section (procedure). |
|  | 20 | ***Field notes – were field notes made during and/or after the interview or focus group?***  Field notes were made both during and after the interviews by BR to inform follow-up questions, subsequent interviews and analyses. |
|  | 21 | ***Duration – what was the duration of the interviews and focus groups?***  See methods section (procedure). |
|  | 22 | ***Data saturation – was data saturation discussed?***  See methods section (participants) |
|  | 23 | ***Transcripts returned – were transcripts returned to participants for comment and/or correction?***  See methods section (procedure). |
| **Domain 3: analysis and findings** | | |
| Data analysis | 24 | ***Number of data coders – how many data coders coded the data?***  See methods section (analysis). |
|  | 25 | ***Description of the coding tree – did authors provide a description of the coding tree?***  A hierarchical coding tree was not developed. |
|  | 26 | ***Derivation of themes – were themes identified in advance or derived from the data?***  Themes were derived from the data. |
|  | 27 | ***Software – what software, if applicable, was used to manage the data?***  See methods section (analysis). |
|  | 28 | ***Participant checking – did participants provide feedback on the findings?***  See methods section (analysis). |
| Reporting | 29 | ***Quotations presented – were participant quotations presented to illustrate themes/findings? Was each quotation identified (e.g. participant number)?***  Illustrative quotations were used and attributed to specific participants. |
|  | 30 | ***Data and findings consistent - was there consistency between the data presented and the findings?***  See results section. |
|  | 31 | ***Clarity of major themes – were major themes clearly presented in the findings?***  See results section, where the major themes are presented. |
|  | 32 | ***Clarity of minor themes – is there a description of diverse cases or discussion of minor themes?***  See results section, where diverse cases and minor themes are discussed. |

Tong, A., Sainsbury, P. and Craig, J. 2007. Consolidated criteria for reporting qualitative research (COREQ): a 32-item checklist for interviews and focus groups. *International Journal for Quality in Health Care.* **19**(6), pp.349-357.
