# Supplementary material for: The understanding, application and influence of complexity in national physical activity policy-making
Source: Health Res Policy Syst. 2022 May 31;20:59. doi: 10.1186/s12961-022-00864-9 (PMC9153223; doi:10.1186/s12961-022-00864-9)
Supplement: Supplementary file 2 — Additional file 2: Statement of sampling and recruitment. [file 12961_2022_864_MOESM2_ESM.docx]

## Recruitment

Participants were invited to participate by email, letter or social media.

## Sampling

A purposive sample was taken from the population of UK national physical activity policy-makers. The exact composition of this population is unknown. Details of civil servants in particular are not always in the public domain. Policy documents and websites were used to identify policy-makers whose remit included physical activity. Following initial purposive sampling, a snowball strategy was employed.

## Representativeness

Approximately one-quarter of the individuals approached volunteered to participate in this study. The 10 participants worked for 8 different government or associated organisations. Physical activity policy-makers represent a very niche group of individuals working within UK government organisations. Therefore, we are confident that our sample reflects a range of perspectives from which our subsequent claims can be made. We acknowledge ‘missing voices’ in our study limitations.

## Differences between recruited and analysed samples

Not applicable. All recruited participants provided data for analysis.
