# Supplementary material for: The understanding, application and influence of complexity in national physical activity policy-making
Source: Health Res Policy Syst. 2022 May 31;20:59. doi: 10.1186/s12961-022-00864-9 (PMC9153223; doi:10.1186/s12961-022-00864-9)
Supplement: Supplementary file 3 — Additional file 3: Interview guide. [file 12961_2022_864_MOESM3_ESM.docx]

# Additional file 3 – Interview guide

- In your experience what are the biggest challenges to physical activity promotion?
- What are the challenges to developing a collaborative cross-government approach to increasing public health and physical activity participation among the general population?
- Some people have described physical inactivity as a complex problem in need of complex solutions. What does complexity mean to you in the context of physical inactivity?
- To what extent do these ideas and the notion of whole-systems approaches influence your work?
- How does academic research evidence inform your work?
- What advice would you give to academic researchers trying to influence physical activity policy?
